# Weighted Gene Co-expression Network Analysis for RNA-Sequencing Data of the Varicose Veins Transcriptome

**DOI:** 10.3389/fphys.2019.00278

**Published:** 2019-03-19

**Authors:** Jianbin Zhang, Qiangqiang Nie, Chaozeng Si, Cheng Wang, Yang Chen, Weiliang Sun, Lin Pan, Jing Guo, Jie Kong, Yiyao Cui, Feng Wang, Xueqiang Fan, Zhidong Ye, Jianyan Wen, Peng Liu

**Affiliations:** ^1^Department of Cardiovascular Surgery, China-Japan Friendship Hospital, Beijing, China; ^2^Department of Cardiovascular Surgery, Peking University China-Japan Friendship School of Clinical Medicine, Beijing, China; ^3^Department of Operations and Information Management, China-Japan Friendship Hospital, Beijing, China; ^4^Department of Medicine, Krannert Institute of Cardiology, Indiana University School of Medicine, Indianapolis, IN, United States; ^5^MOE Key Laboratory of Bioinformatics, Bioinformatics Division and Center for Synthetic and Systems Biology, TNLIST, School of Medicine, Tsinghua University, Beijing, China; ^6^Institute of Clinical Medical Sciences, China-Japan Friendship Hospital, Beijing, China

**Keywords:** weight gene co-expression network analysis, RNA-sequencing, varicose veins, interferon, GBP5, guanylate-binding protein 5

## Abstract

**Objective:**

Varicose veins are a common problem worldwide and can cause significant impairments in health-related quality of life, but the etiology and pathogenesis remain not well defined. This study aims to elucidate transcriptomic regulations of varicose veins by detecting differentially expressed genes, pathways and regulator genes.

**Methods:**

We harvested great saphenous veins (GSV) from patients who underwent coronary artery bypass grafting (CABG) and varicose veins from conventional stripping surgery. RNA-Sequencing (RNA-Seq) technique was used to obtain the complete transcriptomic data of both GSVs from CABG patients and varicose veins. Weighted Gene Co-expression network analysis (WGCNA) and further analyses were then carried out with the aim to elucidate transcriptomic regulations of varicose veins by detecting differentially expressed genes, pathways and regulator genes.

**Results:**

From January 2015 to December 2016, 7 GSVs from CABG patients and 13 varicose veins were obtained. WGCNA identified 4 modules. In the brown module, gene ontology (GO) analysis showed that the biological processes were focused on response to stimulus, immune response and inflammatory response, etc. Kyoto encyclopedia of genes and genomes (KEGG) pathway analysis showed that the biological processes were focused on cytokine-cytokine receptor interaction and TNF signaling pathway, etc. In the gray module, GO analysis showed that the biological processes were skeletal myofibril assembly related. The immunohistochemistry staining showed that the expression of ASC, Caspase-1 and NLRP3 were increased in GSVs from CABG patients compared with varicose veins. Histopathological analysis showed that in the varicose veins group, the thickness of vascular wall, tunica intima, tunica media and collagen/smooth muscle ratio were significantly increased, and that the elastic fiber/internal elastic lamina ratio was decreased.

**Conclusion:**

This study shows that there are clear differences in transcriptomic information between varicose veins and GSVs from CABG patients. Some inflammatory RNAs are down-regulated in varicose veins compared with GSVs from CABG patients. Skeletal myofibril assembly pathway may play a crucial role in the pathogenesis of varicose veins. Characterization of these RNAs may provide new targets for understanding varicose veins diagnosis, progression, and treatment.

## Introduction

Varicose veins are a common problem worldwide, affecting approximately 25% of the population (Callam, 1994; Evans et al., 1999) and causing significant impairments in health-related quality of life (HRQoL) (Smith et al., 1999; Kurz et al., 2001; Eberhardt and Raffetto, 2005). Symptoms in varicose vein patients span from cosmetic worries to severe refractory ulcer (Rabe and Pannier, 2012). Until now, the etiology and pathogenesis of varicose vein remain not well defined (Li et al., 2014).

Transcriptomic alteration of great saphenous vein can lead to vascular wall structure and morphological changes, which may contribute to the pathogenesis of varicose veins. RNA-Sequencing (RNA-Seq) is a relatively novel approach to transcriptome profiling, which can obtain the complete transcriptomic information and allow for more extensive analysis compared with microarray platforms (Wang et al., 2009). Gene expression profiling with co-expression analysis has been widely used in identifying gene expression level in various diseases, such as neurological disorders, cancer and some metabolic disorders (Sundarrajan and Arumugam, 2016). Weighted gene co-expression network analysis (WGCNA) is a powerful method to identify co-expressed groups of genes from large heterogeneous messenger RNA expression data sets (Clarke et al., 2013), and it is widely used to illuminate transcriptomic alterations in some diseases such as cancer and acute aortic dissection (Zhang and Horvath, 2005; Wang et al., 2017). Compared to partial correlation and information theory (PCIT) methods, WGCNA has proven its superiority and it may identify higher-order relationships by focusing on the integrated function of gene modules (Zhao et al., 2010; Kadarmideen and Watson-Haigh, 2012).

In this study, we harvested GSVs from CABG patients and varicose great saphenous veins (GSV) from conventional stripping surgery. RNA-Seq technique was used to obtain the complete transcriptomic data of both GSVs from CABG patients and varicose GSV. WGCNA and further analyses were then carried out with the aim to elucidate transcriptomic regulations of varicose veins by detecting differentially expressed genes, pathways and regulatory genes. We integrated RNA-Seq to associated gene networks and pathways with pathological process in varicose veins.

## Materials and Methods

### Study Population and Specimen Collection

The study population consists of patients who underwent CABG because of coronary artery disease and patients who underwent conventional great saphenous vein stripping surgery because of varicose veins. The GSVs from CABG patients were harvested during the CABG surgery and varicose great saphenous vein samples were harvested during the conventional GSV stripping surgery. The GSV phlebectomy was carried out about 5 cm above the knee and the specimens were 2 cm in length. When harvested, the surrounding tissue was removed and each specimen was divided into 2 segments, one stored in liquid nitrogen with a frozen tube and the other one in 10% formalin solution, both immediately. This study was carried out in accordance with the recommendations of the ethics committee of China-Japan Friendship Hospital with written informed consent from all subjects. All subjects gave written informed consent in accordance with the Declaration of Helsinki. The protocol was approved by the ethics committee of China-Japan Friendship Hospital.

### Histopathological Analysis of Varicose Veins

The GSVs from CABG patients and varicose veins were fixed in 10% formalin for at least 24 h. Specimens were dehydrated, embedded with paraffin and sectioned (Tissue-Tek and IVS-410, Sakura Company, Japan) for further investigations. Hematoxylin-Eosin (HE), Masson trichrome and elastic fiber staining for morphologic analysis were carried out for both GSVs from CABG patients and varicose veins. We measured the thickness at 4 points around the vessel circumference, including 12-o’clock, 3-o’clock, 6-o’clock and 9-o’clock. Then, the histopathological analysis was carried out under an optical microscope (DM4000 B, Leica, German) with Image J software (Version 1.48). The person who performed the histopathological examination was blinded to whether the specimens were GSVs from CABG patients or varicose veins. The immunohistochemistry staining for ASC, Caspase-1 and NLRP3 (NOD-like receptor family, pyrin domain-containing 3) was carried out with antibodies for human ASC (1:200, Proteintech, IL, United States), Caspase-1 (1:200, Proteintech, IL, United States) and NLRP3 (1:100, Abcam, Cambridge, United Kingdom) using EnVison method. Brown or brown-black particles in the cytoplasm were considered as positive expressions.

### RNA Isolation and Quality Examination

RNA was isolated with TRIzol Reagent (Life Technologies, CA, United States). RNA degradation and contamination were detected by 1% agarose gels electrophoresis. RNA purity was checked using the Qubit^®^ 3.0 Fluorometer (Life Technologies, CA, United States). RNA integrity and concentration were assessed using the RNA Nano 6000 Assay Kit of the Bioanalyzer 2100 system (Agilent Technologies, CA, United States).

### Library Preparation, Construction, and Examination

A total amount of 2 μg RNA per sample was used as input material for the RNA sample preparations. Sequencing libraries were generated using NEBNext^®^ Ultra^TM^ RNA Library Prep Kit for Illumina^®^ (#E7530L, NEB, United States) following the manufacturer’s recommendations. Index codes were added to attribute sequences to each sample. Briefly, mRNA was purified from total RNA using poly-T oligo-attached magnetic beads. Fragmentation was carried out using divalent cations under elevated temperature in NEBNext First Strand Synthesis Reaction Buffer (5X). First strand cDNA was synthesized using random hexamer primer and RNase H. Second strand cDNA synthesis was subsequently performed using buffer, dNTPs, DNA polymerase I and RNase H. The library fragments were purified with QIAquick PCR kits and elution with EB buffer, subsequently, terminal repair, A-tailing and adapter were implemented. The aimed products were retrieved by agarose gel electrophoresis and PCR was performed. Then the library was completed.

The main procedure for library construction included mRNA concentration, fragmentation, cDNA synthesis, end repairing, add A-tailing and adapter, fragments selection and library purification. The reagent used included NEBNext super speed RNA Library Prep Kit for Illumina; Beckman AM Pure XP beads, Beckman; 80% Ethanol; Promega magnetic frame PCR instrument, ABI 9700.

RNA concentration of library was measured using Qubit^®^ RNA Assay Kit in Qubit^®^ 3.0 to preliminary quantify and then dilute to 1 ng/μl. Insert size was assessed using the Agilent Bioanalyzer 2100 system (Agilent Technologies, CA, United States), and qualified insert size was accurately quantized using StepOnePlus^TM^ Real-Time PCR System (Library valid concentration >10 nM).

### Library Clustering, Sequencing, and Quality Control

The clustering of the index-coded samples was performed on a cBot cluster generation system using HiSeq PE Cluster Kit v4-cBot-HS (Illumina) according to the manufacturer’s instructions. After cluster generation, the libraries were sequenced on an Illumina Hiseq 4000 platform and 150 bp paired-end reads were generated. The Sequencing quality was controlled by MultiQC tool (Ewels et al., 2016).

### RNA-Seq Data Normalization and Differentially Expressed Genes (DEGs) Selection

The number of reads mapped to each gene was counted using HTSeq v0.6.0. Fragments per kilobase of exon per million reads mapped (FPKM) was then calculated based on length of the gene and reads mapped to the gene. Then, raw RNA-Seq data of GSVs from CABG patients and varicose veins was subjected to DEGs analysis. DEGs were identified by DESeq2 (Love et al., 2014) package in R language. We applied false discovery rate (FDR) criterion proposed by Benjamini and Hochberg (Reiner-Benaim, 2007) for multiple testing corrections of the raw *P*-value. The threshold of DEGs was set as FDR < 0.05.

### Weighted Gene Co-expression Network Analysis

We selected genes for network analysis based on their variation (*SD* > 0.25) and built the co-expression network using WGCNA package in R language (Langfelder and Horvath, 2008). Pearson’s correlations between each gene were calculated to build an adjacency matrix. The power parameter (β) was set to 14 based on the scale-free topology criterion (Zhang and Horvath, 2005). Then the topological overlap measure (TOM) and corresponding dissimilarity (1-TOM) were calculated using adjacency matrix. (1-TOM) was used as a distance for gene hierarchical cluster, and then DynamicTree Cut algorithm (Langfelder et al., 2008) was used to identify the modules (defined as clusters of highly interconnected genes). Each module has a different color. We use Z-score summary predicted by the module preservation function implemented in the WGCNA package to assess the module conservation. The first principle gene in the module was defined as module eigengene (ME). Then, we used Pearson’s correlation between expression profile of each gene and ME to identify the module membership (MM). The summing of connectivity for one gene with other genes in the module was defined as intra-module connectivity (Kin).

### Module-Traits Relationships and Functional Categorization of Modules

Using the ME, the module-traits relationships were estimated by calculating the Pearson’s correlations between the ME and the traits of interest. Those module-traits relationships were used to select potential biologically interesting modules for downstream analysis. Modules were selected when they had a correlation >0.5 with at least one of the selected traits. Genes in the module were selected when their intra-modular connectivity with that particular module was >0.6, and the intra-modular connectivity with all other modules <0.6. The intra-modular connectivity is calculated as the correlation between the gene’s expression profile and the expression profile of the ME. Another gene characteristic is the gene trait correlation: the correlation between the gene’s expression profile and the phenotype of interest. The uniqueness of the 4 modules was characterized based on gene ontology (GO), including the biological process, cellular components and molecular functions using clusterProfiler packages (Yu et al., 2012).

### Statistical Analysis

Continuous variables were present as mean and standard deviation. Discrete variables were present as percentages. For continuous variables, we carried out the normality test. For normally distributed variables, we performed independent *t*-test. For non-normally distributed variables, we performed a non-parametric test. For discrete variables, the chi-square test or Fisher’s exact test were performed. Data analysis was performed using SPSS version 22 (SPSS Inc., Chicago, IL, United States) and R software version 3.2.3 (package “WGCNA” “DESeq2”). A *P*-value of <0.05 was considered statistically significant.

### Availability of Supporting Data

The RNA-Seq data in the study can be found in NCBI’s Sequence Read Archive (SRA) accession: PRJNA523048 (https://www.ncbi.nlm.nih.gov/Traces/study/?acc=PRJNA523048).

## Results

### Demographics and Clinical Features of the Patients

From January 2015 to December 2016, 7 GSVs from CABG patients and 13 varicose GSV were obtained. In the CABG group, there were 5 males and 2 females, with the mean age being 62.9 years old. In the varicose vein group, there were 10 males and 3 females, with the mean age being 55.7 years old. The clinical features of the patients are shown in Table 1.

**Table 1:** Demographics and clinical features of the patients.

	GSVs from CABG patients (7)	Varicose veins (13)	*P*-value
Age	62.9 ± 10.4	55.7 ± 10.9	0.166
Gender (Male/Female)	5/2	10/3	0.999
BMI	25.2 ± 4.1	25.4 ± 3.0	0.904
Hypertension	5	3	0.062
Diabetes mellitus	4	2	0.122
Hyperlipidemia	3	1	0.101
Smoker	2	1	0.270


*GSV, great saphenous vein; CABG, coronary artery bypass grafting; BMI, body mass index.*

### Global Identification of Differentially Expressed RNAs in Varicose Veins

**FIGURE 1:**
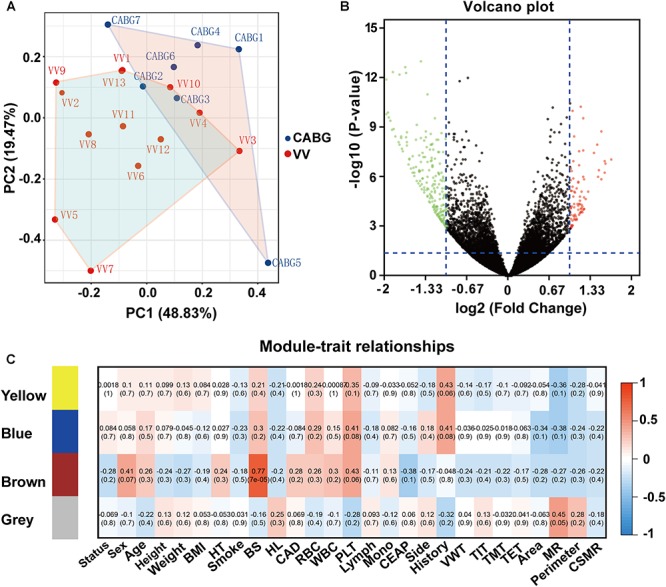
Differentially expressed genes (DEGs) analysis of varicose veins (VV) and great saphenous veins (GSV) from coronary artery bypass grafting (CAGB) patients. **(A)** PCA plot of all the samples. The VV group and CABG group show distinct separation. **(B)** Volcano plot of differentially expressed genes of VV vs. CABG. Green dots indicate downregulated genes, red dots indicate upregulated genes, and black dots indicate non-significance with cut-off criteria (*P*-value <0.05, abs (log2FC) >1). **(C)** DEGs were divided into four module genes with WGCNA analysis. The heatmap visualization of correlation between module genes and samples traits.

We characterized global transcriptional RNA expression between GSVs from CABG patients and those from varicose veins. Before alignment, low-quality reads and those containing adapter or poly-N were removed using MultiQC. The remaining reads were mapped to the assembly hg19 genome using the default parameters in STAR (v2.5.1b) aligner. This resulted in an average of 19033962 uniquely mapped reads per sample, of which an average of 97.56% were mapped in the intragenic region (within introns or exons). On average 19572270 reads were detected among the mapped reads. Read counts were estimated at the gene level using HTSeq. Finally we identified 333 differentially expressed genes, including 87 up-regulated and 246 down-regulated (Supplementary Data Sheet S1). The volcano plot in Figure 1 showed the differentially expressed genes.

### Weight Gene Co-expression Network Analysis (WGCNA)

Weight gene co-expression network analysis approach was applied to the data count obtained from RNA-Seq of 7 GSVs from CABG patients and 13 varicose vein samples. We used the differentially expressed genes to construct the co-expression network. Gene modules (clusters of highly co-expressed genes) were detected and assigned different colors. Finally, we identified 4 modules containing 285 genes, labeled by yellow, brown, blue and gray (Figure 1). Among these modules, there were yellow modules containing 41 genes, brown module containing 143 genes, gray module containing 40 genes, blue module containing 61 genes.

### Module-Trait Relationship Analysis

In these identified modules, an eigengene was calculated. Then, we correlated the selected traits and modules’ eigengene to calculate the module-traits relationships (Figure 1). The results showed that several modules were highly correlated with one or more selected traits. The most relevant in module-trait relationship analysis was found between gray module and status of the sample (*r* = 0.92, *P* = 1 × 10^-8^). Therefore, for further analyses, we selected the gray module as a module of interest. The gray module was also associated with CEAP (Clinical, Etiological, Anatomical and Pathological) categories, the presence of coronary artery disease, side of the great saphenous vein, vascular wall thickness and collagen/smooth muscle ratio.

### Functional Enrichment of Modules

**FIGURE 2:**
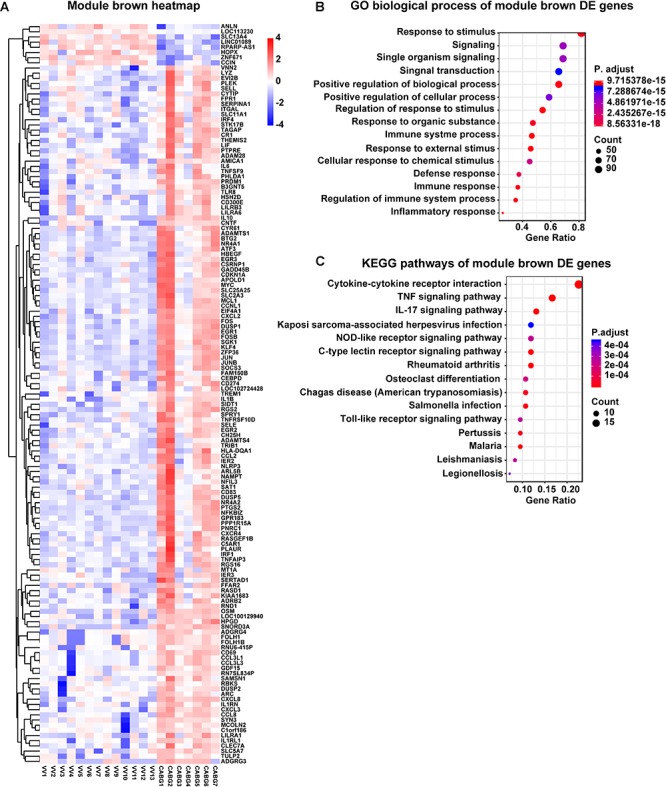
Gene functional analysis of module brown genes. **(A)** Gene expression of module brown genes with RNA-seq data. **(B)** Gene ontology analysis and **(C)** Kyoto encyclopedia of genes and genomes (KEGG) pathway analysis of module brown genes.

**FIGURE 3:**
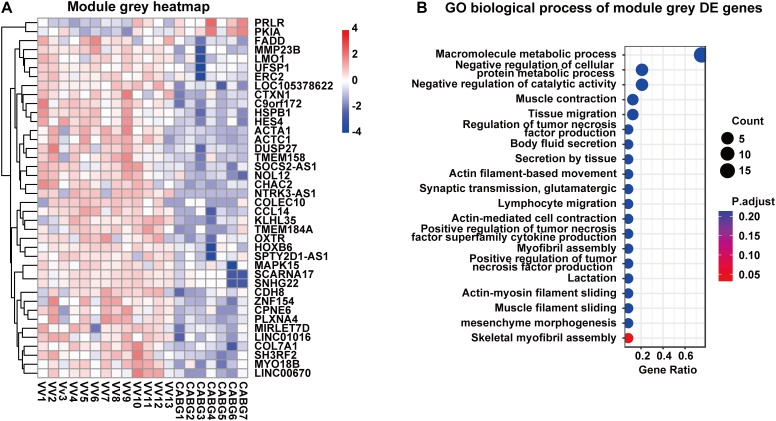
Gene functional analysis of module gray genes. **(A)** Gene expression of module gray genes with RNA-seq data. **(B)** Gene ontology analysis of module gray genes.

Genes in the modules were subjected to GO functional analysis and KEGG (Kyoto encyclopedia of genes and genomes) pathway analysis. Gene functional analysis of DE genes can be found in Supplementary Figure S1. In the brown module, GO analysis showed that the biological processes were enriched in response to stimulus, immune response and inflammatory response, etc. KEGG pathway analysis demonstrated that the difference expression genes may function through cytokine-cytokine receptor interaction and TNF signaling pathway (Figure 2). In the gray module, GO analysis showed that the biological processes were skeletal myofibril assembly related (Figure 3). Gene functional analysis of module yellow genes can be found in Supplementary Figure S2.

### Immunohistochemistry Staining of ASC, Caspase-1 and NLRP3 Expression

**FIGURE 4:**
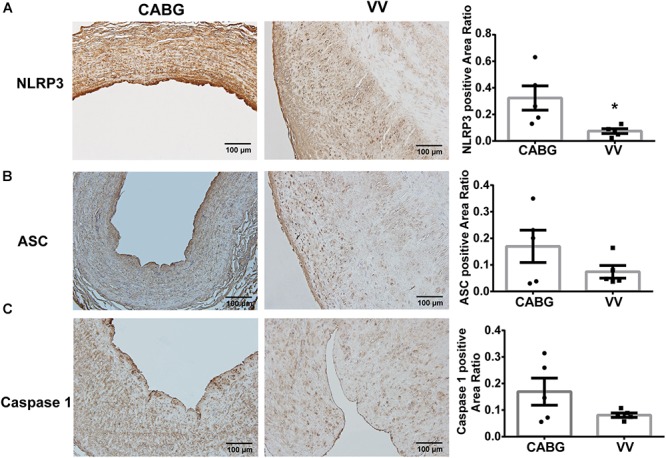
The immunohistochemistry staining showed that the expression of NLRP3, ASC and Caspase 1 were decreased in varicose veins. **(A)** Representative photomicrographs of immunohistochemistry staining for NLRP3. Box plot showed that the expression of NLRP3 significantly decreased in varicose veins. **(B)** Representative photomicrographs of immunohistochemistry staining for ASC. Box plot showed that the expression of ASC has a trend to decrease in varicose veins. **(C)** Representative photomicrographs of immunohistochemistry staining for Caspase 1. Results show a trend to decrease in varicose veins. ^∗^*P* < 0.05.

The immunohistochemistry staining showed that the NLRP3 expression in varicose veins was decreased compared with GSVs from CABG patients (Figure 4). The expression of Caspase-1 and ASC had a downward tendency, but the difference was not statistically significant (Figure 4).

### Histopathological Characteristics of the Samples

**FIGURE 5:**
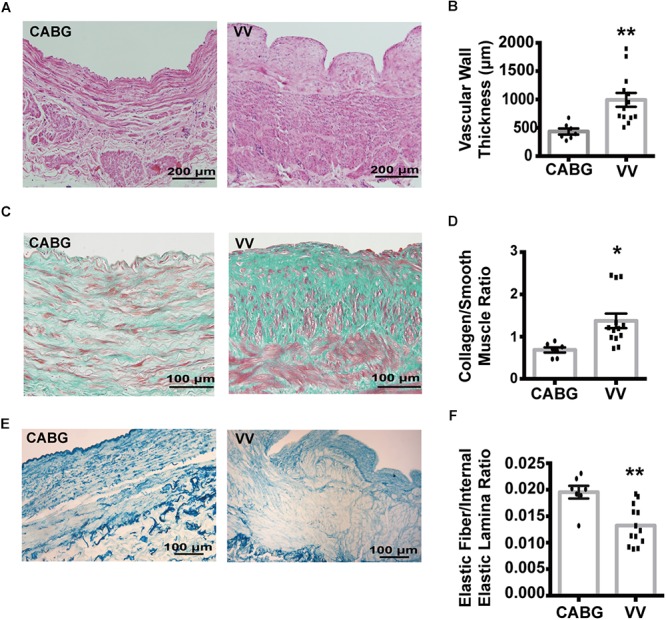
The thickness of vascular wall, tunica intima, tunica media and collagen/smooth muscle ratio were significantly increased, the elastic fiber/internal elastic lamina ratio was decreased in varicose veins. **(A)** Representative photomicrographs of hematoxylin-eosin staining for vascular wall thickness. **(B)** Box plot showed that vascular wall thickness was significantly increased in varicose veins. **(C)** Representative photomicrographs of Vitoria blue staining for elastic fiber. **(D)** Box plot showed that the elastic fiber/internal elastic lamina ratio was decreased in varicose veins. **(E)** Representative photomicrographs of Masson staining for vascular smooth muscle and collagen. **(F)** Box plot showed that the collagen/smooth muscle ratio were significantly increased. ^∗∗^*P* < 0.01; ^∗^*P* < 0.05.

The histopathological analysis showed that in varicose veins, the thickness of vascular wall, tunica intima, tunica media and the collagen/smooth muscle ratio were significantly increased, and the ratio of elastic fiber/internal elastic lamina was decreased (Figure 5). The details are shown in Table 2.

**Table 2:** Histopathologic characteristics of GSVs from CABG patients and varicose veins.

	GSVs from CABG patients (7)	Varicose veins (13)	*P*-value
VWT (μm)^∗∗^	435.0 ± 141.4	992.6 ± 441.5	0.005
TIT (μm)^∗^	67.8 ± 22.8	247.6 ± 166.8	0.012
TMT (μm)^∗∗^	212.8 ± 49.0	519.1 ± 221.6	<0.001
TET (μm)	154.4 ± 96.5	226.0 ± 142.9	0.253
Lemen area (mm^2^)	2.1 ± 1.4	2.6 ± 1.2	0.211
MD (mm)	4.3 ± 1.9	3.5 ± 1.0	0.255
Perimeter (mm)	9.2 ± 4.0	8.8 ± 3.0	0.757
CSMR^∗^	0.69 ± 0.16	1.37 ± 0.63	0.001
EFIEL^∗∗^	0.020 ± 0.003	0.013 ± 0.004	0.001


*GSV, great saphenous vein; CABG, coronary artery bypass grafting; VWT, vascular wall thickness; TIT, tunica intima thickness; TMT, tunica media thickness; TET, tunica externa thickness; MD, maximum diameter; CSMR, collagen/smooth muscle ratio; EFIEL, elastic fiber/internal elastic lamina ratio; ^∗^*P* < 0.05; ^∗∗^*P* < 0.01.*

## Discussion

In the present study, we carried out WGCNA based on RNA-Seq data, clinical information and the histopathologic characteristics of GSVs from CABG patients and varicose veins from conventional surgery and identified several candidate genes and biological processes which may contribute to the pathogenesis of varicose veins.

It is commonly considered that weakness of venous wall, venous valve dysfunction, and increased intravenous pressure are the main causes of varicose veins (Meissner et al., 2007; Oklu et al., 2012). Though venous valve dysfunction and increased intra-venous pressure were thought to be the initiating factors, in clinical practice, some varicose vein patients have functional venous valves and venous wall distension appear before valve incompetence (Alexander, 1972). The histology of venous walls was reported to be primitive, with incomplete vein wall formation and decreased elastic component in recurrent varicose veins (Brake et al., 2013). These phenomena indicated that the primary variation of venous wall components and structure may be vital in the pathogenesis of varicose veins (Clarke et al., 1992; Elsharawy et al., 2007). Therefore, an increasing number of studies began to focus on the primary changes of venous wall components and structure in patients with varicose veins.

Gomez reported (Gomez et al., 2014) that decreased prostaglandin E2 reduced matrix metalloproteinases-1 activity during vascular wall remodeling and, consequently, increased collagen density in varicose veins. Primary vascular smooth muscle cell (VSMC) variation may be the initiating factor for varicose veins (Venturi et al., 1996), and down-regulation of desmuslin promotes VSMC switch to a synthetic phenotype from a contractile phenotype (Xiao et al., 2009). Desmuslin knockdown also leads to an increase in matrix metalloproteinase-2 and collagen activity (Xiao et al., 2010), which may cause the formation of varicose veins.

Some studies have investigated the differentially expressed genes in primary varicose veins. Kim et al. (2005) recognized 3 cDNAs and Görmüs et al. (2014) found that up-regulation of elastin and related genes may play an important role in the pathogenesis of varicose veins. Cui et al. (2012) carried out microRNAs (miRNAs) profiling in the GSV in patients with chronic venous insufficiency (CVI) and found that miR-34a, miR-155, and miR-202 might play crucial rules in CVI and varicose veins. Li et al. (2014) reported the aberrantly expressed long non-coding RNAs (lncRNAs) involved in varicose veins and predicted their potential functions. Then further investigations (Li et al., 2015) showed that low expressed lncRNA-GAS5 may facilitate VSMC proliferation and migration through Annexin A2, which may promote the pathogenesis of varicose vein. Anwar et al. (2017) applied nuclear magnetic resonance spectroscopy and ultra-performance liquid chromatography-mass spectrometry to analyze metabolic profiling of varicose veins, and also reported 4 differentially expressed miRNAs. All the previous studies have investigated some aspects of RNA expression, but few of them have evaluated transcriptomic alterations. RNA-Seq is a relatively novel technique utilizing a high-throughput sequencing method to evaluate transcriptomic alterations, including total RNA, pre-mRNA, and non-coding RNA (Kukurba and Montgomery, 2015). WGCNA is also novel and powerful in identifying co-expression modules and is widely used to distinguish transcriptomic variations. In our study, we applied WGCNA on RNA-Seq data, and further investigations showed that some inflammatory RNA may be down-regulated in varicose veins compared with GSVs from CABG patients. The skeletal myofibril assembly pathway may play a crucial role in the pathogenesis of varicose veins.

NLRP3 inflammasome is a key component mediating sterile inflammation. This is a complex consisting of NLRP3, ASC (apoptosis-associated speck-like protein containing a CARD), and caspase-1. It has been reported to be closely related to vascular dysfunction. NLRP3 inflammasome can be activated by cholesterol crystal and is essential for atherogenesis (Duewell et al., 2010), and has been proven to be required for VSMC calcification in atherosclerosis (Wen et al., 2013). It has been reported that obesity can accelerate vascular endothelial dysfunction via the activation of NLRP3 inflammasome and mitochondrial dysfunction (Liu et al., 2015). Bruder et al. reported that NLRP3 inflammasome plays a central role in aldosterone-induced vascular dysfunction (Bruder-Nascimento et al., 2016), and this complex also contributes to VSMC phenotypic transformation (Sun et al., 2017). It has also been reported that activation of NLRP3 inflammasome is a key determinant of venous thrombosis during hypoxic condition in venous disease (Gupta et al., 2017). In our immunohistochemistry staining, the expression of NLRP3, ASC and caspase-1 was decreased in varicose veins compared with GSVs from CABG patients, except for 1 patient with high blood glucose. In conjugation with the WGCNA results, we speculate that NLRP3 inflammasome expression was associated with high blood glucose and may play a role in the pathogenesis of coronary artery atherosclerosis. As for varicose veins, NLRP3 inflammasome has a downward tendency, which suggested that NLRP3 inflammasome mediated inflammation may not contribute to the pathogenesis of varicose veins. However, GO showed that the skeletal myofibril assembly pathway may be vital in the pathogenesis of varicose veins.

It has been reported that insulin-like growth factor I stimulates myofibril development and decreases smooth muscle α-actin (Donath et al., 1994). In varicose veins, the ratio of collagen and smooth muscle significantly increased. Further investigations of differentially expressed genes in gray module may help to elucidate the underlying mechanism of these changes. Therefore, characterization of these RNAs may provide new targets for understanding varicose veins diagnosis, progression, and treatment.

Our study has some limitations. First, the sample size is relatively small. GSVs from CABG patients are not strictly normal, which may cause a bias. Increased cardiovascular risk in CABG patients might increase the expression of NLRP3 inflammasome markers, since those patients often presented hypertension, diabetes and obesity, which have been associated with NLRP3 inflammasome activation. Second, though WGCNA allows for more extensive analysis, over analysis of RNA-Seq data may lead to type I error. Third, this is a preliminary study, and further studies, such as RT-qPCR, western blotting, gene knocking down/out and overexpression should be carried out to shade light of the underlying mechanisms.

## Conclusion

This study shows that there are clear differences in transcriptomic information between varicose veins and GSVs from CABG patients. Some inflammatory RNAs are down-regulated in varicose veins compared with GSVs from CABG patients. Skeletal myofibril assembly pathway may play a crucial role in the pathogenesis of varicose veins. Characterization of these RNAs may provide new targets for understanding varicose veins diagnosis, progression, and treatment.

## Author Contributions

JZ, JW, and PL designed the study. XF, CW, LP, JG, and WS collected the data. CS, YaC, JK, YiC, FW, and QN analyzed the data. QN, JZ, XF, ZY, and JW wrote the manuscript.

## Conflict of Interest Statement

The authors declare that the research was conducted in the absence of any commercial or financial relationships that could be construed as a potential conflict of interest.

## Supplementary Material

FIGURE S1 Gene functional analysis of DE genes.

FIGURE S2 Gene functional analysis of module yellow genes.

DATA SHEET S1 Differentially expressed genes of VV and GSV from CAGB patients.

## References

[B1] Alexander C. J. (1972). The theoretical basis of varicose vein formation. *Med. J. Aust.* 1 258–261. 5014125 10.5694/j.1326-5377.1972.tb50912.x

[B2] Anwar M. A. Adesina-Georgiadis K. N. Spagou K. Vorkas P. A. Li J. V. Shalhoub J. (2017). A comprehensive characterisation of the metabolic profile of varicose veins; implications in elaborating plausible cellular pathways for disease pathogenesis. *Sci. Rep.* 7:2989. 10.1038/s41598-017-02529-y 28592827 PMC5462754

[B3] Brake M. Lim C. S. Shepherd A. C. Shalhoub J. Davies A. H. (2013). Pathogenesis and etiology of recurrent varicose veins. *J. Vasc. Surg.* 57 860–868. 10.1016/j.jvs.2012.10.102 23343668

[B4] Bruder-Nascimento T. Ferreira N. S. Zanotto C. Z. Ramalho F. Pequeno I. O. Olivon V. C. (2016). NLRP3 inflammasome mediates aldosterone-induced vascular damage. *Circulation* 134 1866–1880. 10.1161/CIRCULATIONAHA.116.024369 27803035

[B5] Callam M. J. (1994). Epidemiology of varicose veins. *Br. J. Surg.* 81 167–173. 10.1002/bjs.1800810204 8156326

[B6] Clarke C. Madden S. F. Doolan P. Aherne S. T. Joyce H. O’Driscoll L. (2013). Correlating transcriptional networks to breast cancer survival: a large-scale coexpression analysis. *Carcinogenesis* 34 2300–2308. 10.1093/carcin/bgt208 23740839

[B7] Clarke G. H. Vasdekis S. N. Hobbs J. T. Nicolaides A. N. (1992). Venous wall function in the pathogenesis of varicose veins. *Surgery* 111 402–408. 1557686

[B8] Cui C. Liu G. Huang Y. Lu X. Lu M. Huang X. (2012). MicroRNA profiling in great saphenous vein tissues of patients with chronic venous insufficiency. *Tohoku J. Exp. Med.* 228 341–350. 10.1620/tjem.228.341 23132275

[B9] Donath M. Y. Zapf J. Eppenberger-Eberhardt M. Froesch E. R. Eppenberger H. M. (1994). Insulin-like growth factor I stimulates myofibril development and decreases smooth muscle alpha-actin of adult cardiomyocytes. *Proc. Natl. Acad. Sci. U.S.A.* 91 1689–1690. 10.1073/pnas.91.5.1686 8127866 PMC43228

[B10] Duewell P. Kono H. Rayner K. J. Sirois C. M. Vladimer G. Bauernfeind F. G. (2010). NLRP3 inflammasomes are required for atherogenesis and activated by cholesterol crystals. *Nature* 464 1357–1361. 10.1038/nature08938 20428172 PMC2946640

[B11] Eberhardt R. T. Raffetto J. D. (2005). Chronic venous insufficiency. *Circulation* 111 2398–2409. 10.1161/01.CIR.0000164199.72440.08 15883226

[B12] Elsharawy M. A. Naim M. M. Abdelmaguid E. M. Al-Mulhim A. A. (2007). Role of saphenous vein wall in the pathogenesis of primary varicose veins. *Interact. Cardiovasc. Thorac. Surg.* 6 219–224. 10.1510/icvts.2006.136937 17669815

[B13] Evans C. J. Fowkes F. G. Ruckley C. V. Lee A. J. (1999). Prevalence of varicose veins and chronic venous insufficiency in men and women in the general population: Edinburgh Vein study. *J. Epidemiol. Commun. Health* 53 149–153. 10.1136/jech.53.3.149 PMC1756838 10396491

[B14] Ewels P. Magnusson M. Lundin S. Käller M. (2016). MultiQC: summarize analysis results for multiple tools and samples in a single report. *Bioinformatics* 32 3047–3048. 10.1093/bioinformatics/btw354 27312411 PMC5039924

[B15] Gomez I. Benyahia C. Louedec L. Leséche G. Jacob M. P. Longrois D. (2014). Decreased PGE2 content reduces MMP-1 activity and consequently increases collagen density in human varicose vein. *PLoS One* 9:e88021. 10.1371/journal.pone.0088021 24505358 PMC3914898

[B16] Görmüs U. Timirci-Kahraman O. Ergen A. Kunt A. T. Isbir S. Dalan A. B. (2014). Expression levels of elastin and related genes in human varicose veins. *Folia Biol.* 60 68–73. 24785109 10.14712/fb2014060020068

[B17] Gupta N. Sahu A. Prabhakar A. Chatterjee T. Tyagi T. Kumari B. (2017). Activation of NLRP3 inflammasome complex potentiates venous thrombosis in response to hypoxia. *Proc. Natl. Acad. Sci. U.S.A.* 114 4763–4768. 10.1073/pnas.1620458114 28420787 PMC5422823

[B18] Kadarmideen H. N. Watson-Haigh N. S. (2012). Building gene co-expression networks using transcriptomics data for systems biology investigations: comparison of methods using microarray data. *Bioinformation* 8 855–861. 10.6026/97320630008855 23144540 PMC3489090

[B19] Kim D. I. Eo H. S. Joh J. H. (2005). Identification of differentially expressed genes in primary varicose veins. *J. Surg. Res.* 123 222–226. 10.1016/j.jss.2004.08.003 15680382

[B20] Kukurba K. R. Montgomery S. B. (2015). RNA sequencing and analysis. *Cold Spring Harb. Protoc.* 2015 951–969. 10.1101/pdb.top084970 25870306 PMC4863231

[B21] Kurz X. Lamping D. L. Kahn S. R. Baccaglini U. Zuccarelli F. Spreafico G. (2001). Do varicose veins affect quality of life? Results of an international population-based study. *J. Vasc. Surg.* 34 641–648. 10.1067/mva.2001.117333 11668318

[B22] Langfelder P. Horvath S. (2008). WGCNA: an R package for weighted correlation network analysis. *BMC Bioinformatics* 9:559. 10.1186/1471-2105-9-559 19114008 PMC2631488

[B23] Langfelder P. Zhang B. Horvath S. (2008). Defining clusters from a hierarchical cluster tree: the dynamic tree cut package for R. *Bioinformatics* 24 719–720. 10.1093/bioinformatics/btm563 18024473

[B24] Li L. Li X. The E. Wang L. J. Yuan T. Y. Wang S. Y. (2015). Low expression of lncRNA-GAS5 is implicated in human primary varicose great saphenous veins. *PLoS One* 10:e0120550. 10.1371/journal.pone.0120550 25806802 PMC4373870

[B25] Li X. Jiang X.-Y. Ge J. Wang J. Chen G. J. Xu L. (2014). Aberrantly expressed lncRNAs in primary varicose great saphenous veins. *PLoS One* 9:e86156. 10.1371/journal.pone.0086156 24497937 PMC3908920

[B26] Liu P. Xie Q. Wei T. Chen Y. Chen H. Shen W. (2015). Activation of the NLRP3 inflammasome induces vascular dysfunction in obese OLETF rats. *Biochem. Biophys. Res. Commun.* 468 319–325. 10.1016/j.bbrc.2015.10.105 26514727

[B27] Love M. I. Huber W. Anders S. (2014). Moderated estimation of fold change and dispersion for RNA-seq data with DESeq2. *Genome Biol.* 15:550. 10.1186/s13059-014-0550-8 25516281 PMC4302049

[B28] Meissner M. H. Gloviczki P. Bergan J. Kistner R. L. Morrison N. Pannier F. (2007). Primary chronic venous disorders. *J. Vasc. Surg.* 46(Suppl. S) 54S–67S. 10.1016/j.jvs.2007.08.038 18068562

[B29] Oklu R. Habito R. Mayr M. Deipolyi A. R. Albadawi H. Hesketh R. (2012). Pathogenesis of varicose veins. *J. Vasc. Interv. Radiol.* 23 33–39; quiz 40. 10.1016/j.jvir.2011.09.010 22030459

[B30] Rabe E. Pannier F. (2012). Clinical, aetiological, anatomical and pathological classification (CEAP): gold standard and limits. *Phlebology* 27(Suppl. 1) 114–118. 10.1258/phleb.2012.012S19 22312077

[B31] Reiner-Benaim A. (2007). FDR control by the BH procedure for two-sided correlated tests with implications to gene expression data analysis. *Biom. J.* 49 107–126. 10.1002/bimj.200510313 17342953

[B32] Smith J. J. Garratt A. M. Guest M. Greenhalgh R. M. Davies A. H. (1999). Evaluating and improving health-related quality of life in patients with varicose veins. *J. Vasc. Surg.* 30 710–719. 10.1016/S0741-5214(99)70110-2 10514210

[B33] Sun H. J. Ren X. S. Xiong X. Q. Chen Y. Z. Zhao M. X. Wang J. J. (2017). NLRP3 inflammasome activation contributes to VSMC phenotypic transformation and proliferation in hypertension. *Cell Death Dis.* 8:e3074. 10.1038/cddis.2017.470 28981106 PMC5680591

[B34] Sundarrajan S. Arumugam M. (2016). Weighted gene co-expression based biomarker discovery for psoriasis detection. *Gene* 593 225–234. 10.1016/j.gene.2016.08.021 27523473

[B35] Venturi M. Bonavina L. Annoni F. Colombo L. Butera C. Peracchia A. (1996). Biochemical assay of collagen and elastin in the normal and varicose vein wall. *J. Surg. Res.* 60 245–248. 10.1006/jsre.1996.0038 8592422

[B36] Wang T. He X. Liu X. Liu Y. Zhang W. Huang Q. (2017). Weighted gene co-expression network analysis identifies FKBP11 as a key regulator in acute aortic dissection through a NF-kB dependent pathway. *Front. Physiol.* 8:1010. 10.3389/fphys.2017.01010 29255427 PMC5723018

[B37] Wang Z. Gerstein M. Snyder M. (2009). RNA-Seq: a revolutionary tool for transcriptomics. *Nat. Rev. Genet.* 10 57–63. 10.1038/nrg2484 19015660 PMC2949280

[B38] Wen C. Yang X. Yan Z. Zhao M. Yue X. Cheng X. (2013). Nalp3 inflammasome is activated and required for vascular smooth muscle cell calcification. *Int. J. Cardiol.* 168 2242–2247. 10.1016/j.ijcard.2013.01.211 23453445

[B39] Xiao Y. Huang Z. Yin H. Lin Y. Wang S. (2009). In vitro differences between smooth muscle cells derived from varicose veins and normal veins. *J. Vasc. Surg.* 50 1149–1154. 10.1016/j.jvs.2009.06.048 19703751

[B40] Xiao Y. Huang Z. Yin H. Zhang H. Wang S. (2010). Desmuslin gene knockdown causes altered expression of phenotype markers and differentiation of saphenous vein smooth muscle cells. *J. Vasc. Surg.* 52 684–690. 10.1016/j.jvs.2010.03.069 20573469

[B41] Yu G. Wang L.-G. Han Y. He Q. Y. (2012). clusterProfiler: an R package for comparing biological themes among gene clusters. *OMICS* 16 284–287. 10.1089/omi.2011.0118 22455463 PMC3339379

[B42] Zhang B. Horvath S. (2005). A general framework for weighted gene co-expression network analysis. *Stat. Appl. Genet. Mol. Biol.* 4:Article17. 10.2202/1544-6115.1128 16646834

[B43] Zhao W. Langfelder P. Fuller T. Dong J. Li A. Hovarth S. (2010). Weighted gene coexpression network analysis: state of the art. *J. Biopharm. Stat.* 20 281–300. 10.1080/10543400903572753 20309759

