# Supplementary figures and images for: Weighted Gene Co-expression Network Analysis for RNA-Sequencing Data of the Varicose Veins Transcriptome
Source: Front Physiol. 2019 Mar 19;10:278. doi: 10.3389/fphys.2019.00278 (PMC6433941; doi:10.3389/fphys.2019.00278)

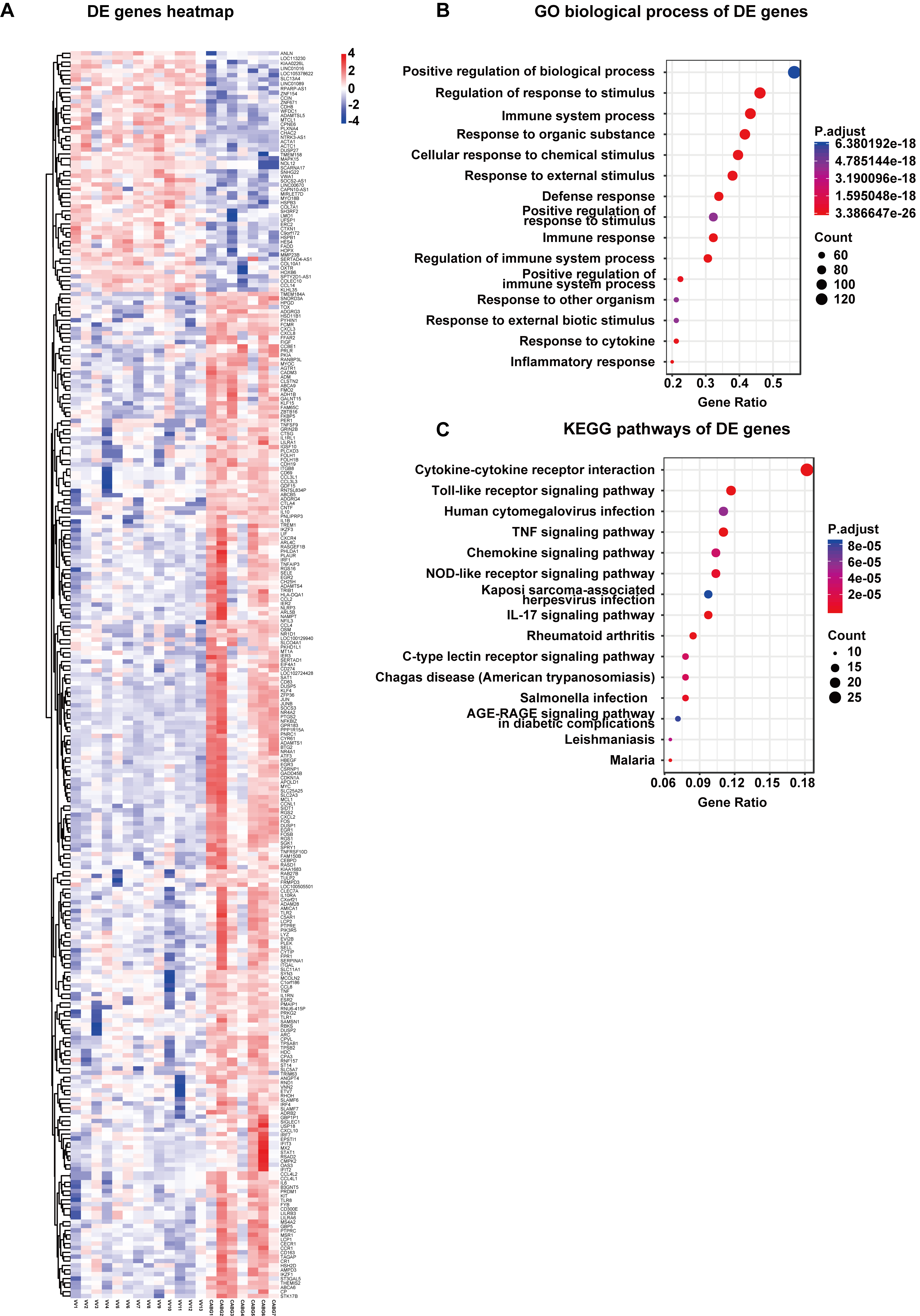

Supplement: FIGURE S1 — Gene functional analysis of DE genes. [file Image_1.TIF]

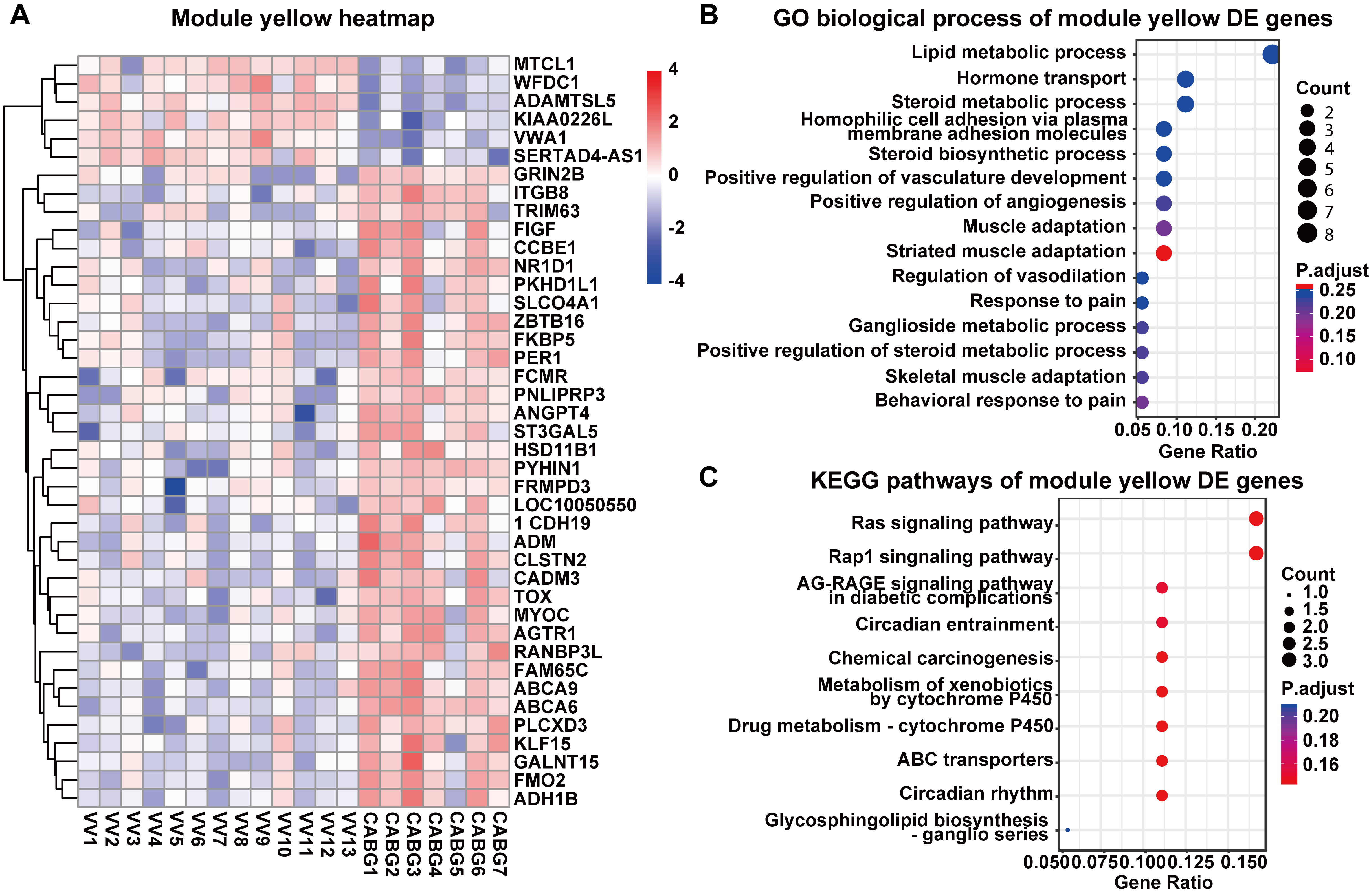

Supplement: FIGURE S2 — Gene functional analysis of module yellow genes. [file Image_2.TIF]
